# Phytochemical Characterization and Potential Anti‐Oxidative Activity of *Lavandula angustifolia* subsp. *pyrenaica* (DC.), *Lavandula x intermedia* Emeric ex Loisel *cv* Grosso, and *cv* Super Essential Oils Compared to a Commercial Lavender Essential Oil

**DOI:** 10.1002/cbdv.202403478

**Published:** 2025-03-04

**Authors:** Eileen Mac Sweeney, Ylenia Pieracci, Vlad Sebastian Popescu, Gianluca Angius, Guido Flamini, Luisa Pistelli, Giulia Abate, Andrea Mastinu

**Affiliations:** ^1^ Department of Molecular and Translational Medicine Division of Pharmacology University of Brescia Brescia Italy; ^2^ Department of Pharmacy University of Pisa Pisa Italy; ^3^ Interdepartmental Research Center “Nutraceuticals and Food for Health” (NUTRAFOOD) University of Pisa Pisa Italy

**Keywords:** antioxidant activity | GC‐MS | lavender essential oils | oxidative stress

## Abstract

This study investigates the phytochemical profiles and antioxidant properties of three lavender essential oils (LEOs) from *Lavandula angustifolia* subsp. *pyrenaica* (DC.), *Lavandula x intermedia* Emeric ex Loisel *cv* Grosso, and *Lavandula x intermedia* Emeric ex Loisel *cv* Super, and compares them to a commercial one. LEOs were extracted by hydrodistillation, analyzed using gas chromatography‐mass spectrometry, and tested for antioxidant effects on human SH‐SY5Y neuroblastoma cells. The major components identified in all the four LEOs were linalool and linalyl acetate; however, in the commercial LEO differences in minor compounds and the presence of additives were found. Antioxidant activity assays revealed significant protection against H₂O₂‐induced oxidative stress for hydrodistilled EOs, while the commercial EO showed no protective effect. Gene expression analysis indicated upregulation of antioxidant‐related genes in cells treated with “Grosso” and “Super” LEOs. This research highlights the potential therapeutic applications of LEOs, with a particular emphasis on the differences between naturally derived oils and commercial variants.

## Graphical abstract

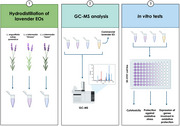

## 1 Introduction

*Lavandula* L. genus, commonly known as lavender, belongs to the Lamiaceae family [1] and includes 39 species as well as hundreds of cultivars and hybrids [2, 3, 4]. Plants belonging to this genus consist mostly of small perennial shrubs; *Lavandula* species can differ in height, color, and shape of their leaves, flowering period, natural habitat, geographical distribution, and essential oils (EOs) composition [4, 5, 6, 7]. Lavender plants commonly reach a height of 30–80 cm [2] and grow in rocky and sunny habitats. They are characterized by linear and semievergreen leaves, presenting a grey‐green color in summer, and becoming silvery during the winter [7]. Lavender plants also exhibit purple flowers, distributed in spike shapes at the end of long stalks [2]. *Lavandula* genus plants are native to the Mediterranean area, including Northern Africa, Turkey, France, Spain, and Italy; in addition, they can also be found in the Canary Islands, Southern Africa, England, India, Australia, and the USA [5, 6, 7]. The most researched and known *Lavandula* species are *Lavandula angustifolia* Miller, also known as *Lavanda vera* or *Lavanda officinalis*, *Lavandula stoechas* L. (French lavender) [6], and *Lavandula latifolia*, commonly known as spike lavender [4, 5, 6, 7]. *L. x intermedia* (lavandin), is a hybrid cross between two natural‐growing lavenders, *L. latifolia* and *L. angustifolia* [4, 5, 6, 7]. *L. angustifolia* subsp. *pyrenaica* (DC.) and two different cultivars of lavandin, namely lavandin Emeric ex Loisel *cv* Grosso and lavandin Emeric ex Loisel *cv* Super, are the focus of this research. *L. angustifolia* is a hardy perennial plant, that can reach a height of 50 cm; this plant is characterized by narrow and linear grey leaves that become greener as they mature, with blue, white, and violet flowers. It can be found mainly in mountainous regions in Italy, France, and Spain at 600–1200 m above sea level, in sunny and drained calcareous soils [5, 7]. *L. latifolia* height can vary from 50 to 100 cm; the leaves are grey and silvery in color and linear to spathulate in shape, with blue to pale purple‐colored flowers; *L. latifolia* has a similar distribution to that of *L. angustifolia*, differing only for the altitude (300 m above sea level) [5, 7]. *L*. “Grosso” and *L*. “Super”, as hybrid plants derived from *L. angustifolia* and *L. latifolia*, grow in the territories the parents naturally habit; these shrubs can reach heights of 60 –150 cm and are characterized by purple to white flowers and grey leaves with shape from linear to spathulate [5]. A noteworthy feature of *L. intermedia*, and hybrid plants in general, is hybrid vigor (also known as heterosis), which means that they exhibit some added emerging benefits compared to their parent plants. In the case of the lavandin subspecies, they are sturdier than true lavender and they contain a higher amount of EOs thanks to their secretory structures being more present than the ones of true lavender [7, 8, 9]. All these characteristics render lavandin cultivars easier and cheaper to cultivate and more profitable as they give more EOs per hectare compared to *L. angustifolia* and other natural lavenders, making them ideal for commercial uses. However, since *L. intermedia* is a cross‐hybrid, is considered sterile [4, 5, 6, 7]. Lavender plants share similar ethnobotanical properties and the main EOs compounds are conserved throughout the whole genus [4, 5, 6, 7]. Since the Middle Ages, lavender and its extracts have been used in the perfume industry, for aromatherapy, skincare, sleep aid, and washing clothes [10, 11]. Other traditional uses include the treatment of respiratory disorders, such as cough, cold, asthma, and sinus congestion [10, 11]. Lavender EOs have been proven to have many pharmacological applications, including anti‐inflammatory and antioxidant properties, along with antimicrobial, antifungal, and antiviral activity [11, 12, 13, 14, 15].

To the best of our knowledge, only a few studies have compared different *Lavandula* species with each other, especially considering *L. intermedia* cultivars. The main objective of this research is to analyze the phytochemical profile of three different lavender EOs (LEOs) (*L. angustifolia*, *L*. “Grosso”, and *L*. “Super”) and to characterize them from a biological point of view while comparing them to a commercially available LEO.

The three LEOs were obtained by hydrodistillation, and their phytochemical content was analyzed by means of gas chromatography‐mass spectrometry (GS‐MS) analysis. The antioxidant activity was evaluated using a human neuroblastoma cell line (SH‐SY5Y).

## 2 Results

### 2.1 LEOs' Phytochemical Profile

The LEOs' chemical composition was analyzed by means of GC‐MS. Specifically, for *L. angustifolia*, the main compounds found were linalool (21.8%) and linalyl acetate (20.1%) (Table 1), belonging to oxygenated monoterpenes, which interestingly represented the most abundant class of compounds found in this EO (71%), followed by oxygenated sesquiterpenes (13.3%), sesquiterpene hydrocarbons (7.9%), monoterpene hydrocarbons (5.7%), and other non‐terpene derivatives (1%) (Table 1).

**TABLE 1:** Chemical composition of *L. angustifolia*, *L*. “Grosso”, *L*. “Super”, and commercially available lavender essential oils (LEOs).

			Relative abundance (%) ± Standard deviation			
Compounds	Class	Linear Retention index	*L. angustifolia*	*L. “Grosso”*	*L. “Super”*	Commercially available LEO
α‐pinene	mh	933	0.2 ± 0.00	0.3 ± 0.01	0.5 ± 0.16	—
Camphene	mh	948	0.2 ± 0.00	0.2 ± 0.01	0.3 ± 0.11	—
1‐octen‐3‐ol	nt	976	—	—	—	0.2 ± 0.01
β‐pinene	mh	977	0.2 ± 0.01	0.3 ± 0.01	0.4 ± 0.17	—
3‐octanone	nt	985	0.2 ± 0.04	—	—	0.2 ± 0.00
Myrcene	mh	991	0.5 ± 0.00	0.4 ± 0.01	0.5 ± 0.15	0.2 ± 0.00
δ‐3‐carene	mh	1011	0.1 ± 0.01	—	—	0.2 ± 0.00
1‐hexyl acetate	nt	1012	0.3 ± 0.02	0.2 ± 0.02	—	—
p‐cymene	mh	1024	0.1 ± 0.01	—	0.2 ± 0.23	—
Limonene	mh	1029	0.5 ± 0.01	0.5 ± 0.04	0.7 ± 0.09	0.4 ± 0.03
1,8‐cineole	om	1031	1.6 ± 0.09	3.9 ± 0.05	6.2 ± 1.46	4.3 ± 0.11
(Z)‐β‐ocimene	mh	1036	2.0 ± 0.22	0.5 ± 0.01	0.8 ± 0.36	1 ± 0.04
(E)‐β‐ocimene	mh	1047	2.1 ± 0.09	0.5 ± 0.04	0.3 ± 0.10	0.3 ± 0.00
γ‐terpinene	mh	1058	—	—	0.4 ± 0.11	—
*cis*‐linalool oxide (furanoid)	om	1072	0.2 ± 0.03	0.1 ± 0.00	—	—
Terpinolene	mh	1089	0.3 ± 0.03	0.3 ± 0.00	12.7 ± 12.41	—
Linalool	om	1101	21.8 ± 1.15	27.0 ± 0.42	19.6 ± 9.45	28.6 ± 0.58
1‐octen‐3‐yl acetate	nt	1113	0.5 ± 0.05	0.1 ± 0.01	—	0.3 ± 0.01
*cis*‐p‐menth‐2‐en‐1‐ol	om	1122	—	—	—	0.2 ± 0.01
2‐p‐Menthen‐1‐ol	om	1126	—	—	—	0.3 ± 0.01
Camphor	om	1145	0.5 ± 0.04	6.4 ± 0.05	5.9 ± 1.89	4.4 ± 0.02
Hexyl isobutyrate	nt	1149	—	0.2 ± 0.03	—	—
Isoborneol	om	1157	—	—	—	1.9 ± 0.03
Borneol	om	1165	2.1 ± 0.22	4.7 ± 0.11	3.3 ± 0.97	4.7 ± 0.04
Lavandulol	om	1166	0.8 ± 0.1	—	—	—
4‐terpineol	om	1177	4.4 ± 0.36	4.3 ± 0.12	3.6 ± 0.44	0.2 ± 0.00
Cryptone	om	1186	0.5 ± 0.02	—	—	0.1 ± 0.00
α‐terpineol	om	1191	4.9 ± 0.18	3.7 ± 0.34	2.6 ± 2.20	1.7 ± 0.02
Hexyl butyrate	nt	1193	—	—	—	0.4 ± 0.01
Nerol	om	1228	0.7 ± 0.08	0.5 ± 0.00	0.7 ± 0.23	—
Cumin aldehyde	om	1239	0.1 ± 0.01	—	—	—
Geraniol	om	1254	1.9 ± 0.23	1.3 ± 0.17	9.5 ± 6.72	—
Linalyl acetate	om	1257	20.1 ± 0.24	19.1 ± 0.23	10.2 ± 5.36	36.1 ± 0.03
Bornyl acetate	om	1286	0.2 ± 0.04	—	—	—
Lavandulyl acetate	om	1292	6.4 ± 0.28	3.0 ± 0.11	2.7 ± 1.69	2.9 ± 0.04
Hexyl tiglate	nt	1332	—	0.3 ± 0.05	—	—
Neryl acetate	om	1365	1.5 ± 0.04	1.1 ± 0.00	1.6 ± 0.64	0.3 ± 0.01
Geranyl acetate	om	1385	3.1 ± 0.13	2.5 ± 0.09	2.1 ± 0.10	1.5 ± 0.07
β‐caryophyllene	sh	1419	5.5 ± 0.10	1.4 ± 0.07	0.6 ± 0.49	7.2 ± 0.37
*trans*‐α‐bergamotene	sh	1436	—	—	—	0.2 ± 0.00
α‐humulene	sh	1453	0.2 ± 0.01	—	—	—
(E)‐β‐farnesene	sh	1458	1.1 ± 0.04	0.9 ± 0.01	0.5 ± 0.12	0.5 ± 0.03
Lavandulyl butyrate	om	1460	—	—	0.1 ± 0.02	—
Germacrene D	sh	1481	0.5 ± 0.07	0.7 ± 0.00	0.7 ± 0.20	0.3 ± 0.02
Lavandulyl isovalerate	om	1508	—	0.4 ± 0.01	0.4 ± 0.05	—
*trans*‐γ‐cadinene	sh	1514	0.7 ± 0.04	—	0.5 ± 0.13	0.3 ± 0.04
Caryophyllene oxide	os	1582	5.3 ± 0.62	0.4 ± 0.01	0.5 ± 0.09	0.4 ± 0.05
1,10‐di‐epi‐cubenol	os	1615	0.4 ± 0.00	—	0.3 ± 0.01	—
T‐cadinol	os	1641	6.8 ± 0.41	2.2 ± 0.10	3.4 ± 0.16	0.3 ± 0.03
α‐Cadinol	os	1654	—	—	0.1 ± 0.02	—
14‐hydroxy‐9‐epi‐(E)‐caryophyllene	os	1670	0.3 ± 0.04	—	—	—
α‐bisabolol	os	1685	—	11.5 ± 0.21	7.3 ± 1.03	—
*cis*‐14‐nor‐muurol‐5‐en‐4‐one	os	1686	0.5 ± 0.02	—	—	—
(Z,E)‐farnesol	os	1689	—	0.5 ± 0.04	0.4 ± 0.03	—
Benzyl benzoate	nt	1763	0.1 ± 0.01	—	—	—
						
Total identified (%)			98.9	99.9	99.9	99.3
Oxygenated monoterpenes			71.0	78.2	68.7	87.1
Sesquiterpenes hydrocarbons			7.9	2.9	2.4	8.4
Monoterpenes hydrocarbons			5.7	2.9	16.9	2.0
Oxygenated sesquiterpene			13.3	14.5	11.9	0.7
Non‐terpene derivatives			1.0	1.4	—	1.1

Abbreviations: mh = monoterpene hydrocarbons, nt = non‐terpene derivatives, om = oxygenated monoterpenes, os = oxygenates sesquiterpenes, sh = sesquiterpene hydrocarbons.

Linalool (27%) and linalyl acetate (19.1%) were, also, the major components of *L*. “Grosso” EO (Table 1), and even in this case, the major detected class of compounds was oxygenated monoterpenes, representing 78.2%, besides oxygenated sesquiterpenes (13.3%), sesquiterpene hydrocarbons (7.9%), monoterpene hydrocarbons (5.7%), and other non‐terpene derivatives (1%) (Table 1).

Interestingly, the chemical profile of *L*. “Super” EO showed that the main components were the oxygenated monoterpenes linalool (19.6%) and linalyl acetate (10.2%), and the monoterpene hydrocarbon terpinolene (12.7%) (Table 1). The main class of components was, also in this case, oxygenated monoterpenes, accounting for 68.7%; unlike the previously described EOs, monoterpene hydrocarbons were the second class, representing 16.9%, followed by oxygenated sesquiterpenes (11.9%), and sesquiterpene hydrocarbons (2.4%), (Table 1).

Finally, the chemical composition of the commercially available LEO was analyzed. The main compounds were linalyl acetate (36.1%) and linalool (28.6%) (Table 1). Interestingly, compounds like 2‐*p*‐menthen‐1‐ol, *cis*‐*p*‐menth‐2‐en‐1‐ol, and 1‐octen‐3‐ol, not present in the previous three LEOs, were also identified. Once again, oxygenated monoterpenes represented the great majority of the chemical composition (87.1%), followed by sesquiterpene hydrocarbons (8.4%), monoterpene hydrocarbons (2%), other non‐terpene derivatives (1.1%), oxygenated sesquiterpenes (0.7%), and phenylpropanoids (0.5%) (Table 1).

### 2.2 LEOs Cytocompatibility

**FIGURE 1:**
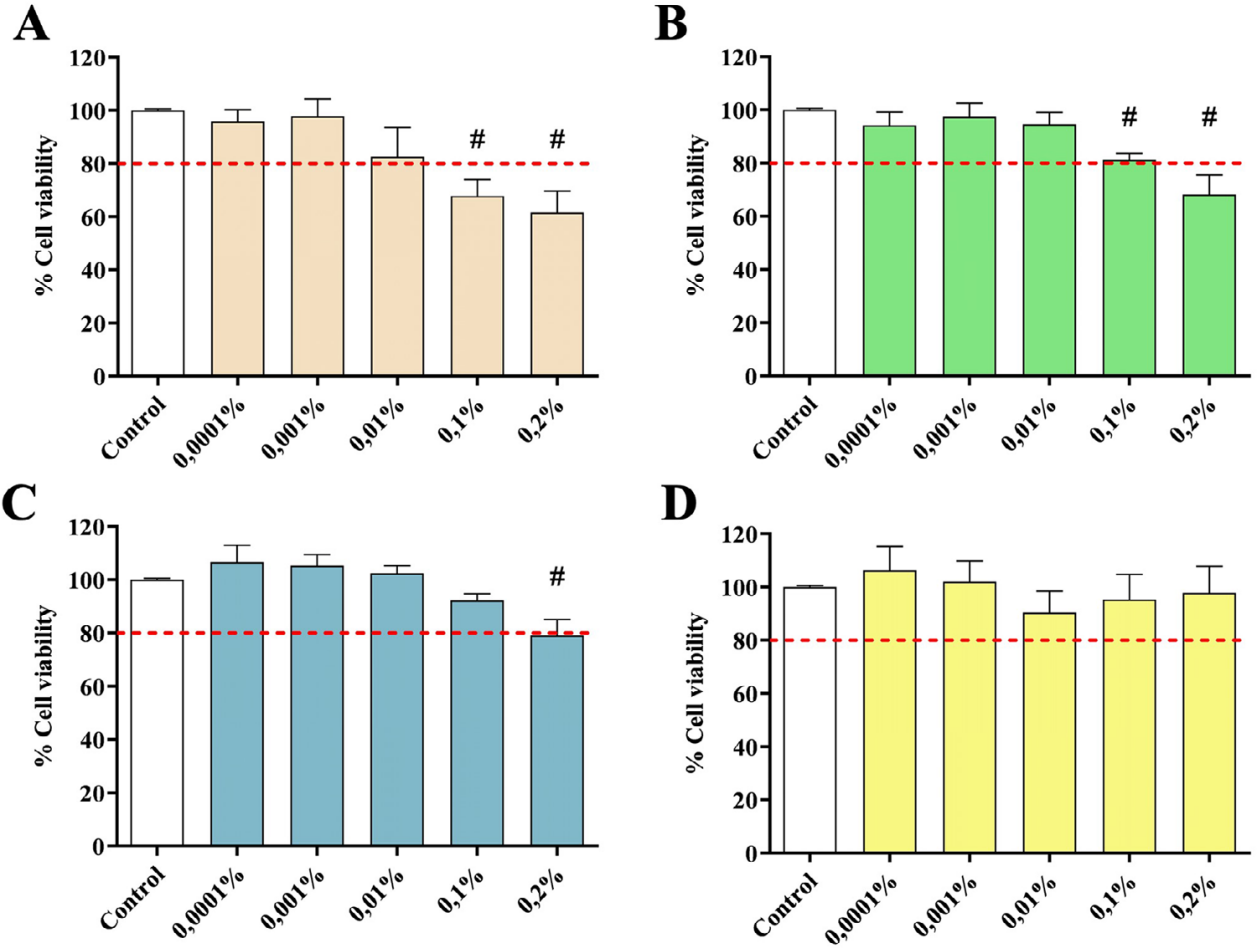
SH‐SY5Y cell line viability after treatment with *L. angustifolia* essential oil (A); *L*. “Grosso” essential oil (B); *L*. “Super” essential oil (C); commercial lavender essential oil (D). Data are presented as a percentage of cell viability compared to untreated cells used as a control. The red dotted line indicates the percentage of cell viability under which the treatments were considered cytotoxic. Data are shown as mean ± SEM of three replicates: ^#^ *p* < 0.05 versus the control group.

The cytocompatibility of *L. angustifolia*, *L*. “Grosso”, *L*. “Super” EOs, and the commercially available LEO was established on the SH‐SY5Y cell line by performing a 3‐[4,5‐dimethylthiazol‐2‐yl]‐2,5‐diphenyl‐tetrazolium bromide (MTT) assay. Cells were treated with the four LEOs at concentrations ranging from 0.0001% to 0.2% for 48 h. Treatments were considered cytotoxic if they reduced cell viability by more than 80%. As shown in Figures 1A,B, the treatment with *L. angustifolia* and *L*. “Grosso” EOs resulted to be cytotoxic at concentrations above 0.1%; considering *L*. EO, only the treatment with the 0.2% concentration significantly reduced cell viability below 80% (Figure 1C). No cytotoxicity was detected for the treatment with the commercially available LEO (Figure 1D).

### 2.3 LEOs Anti‐Oxidative Potential

**FIGURE 2:**
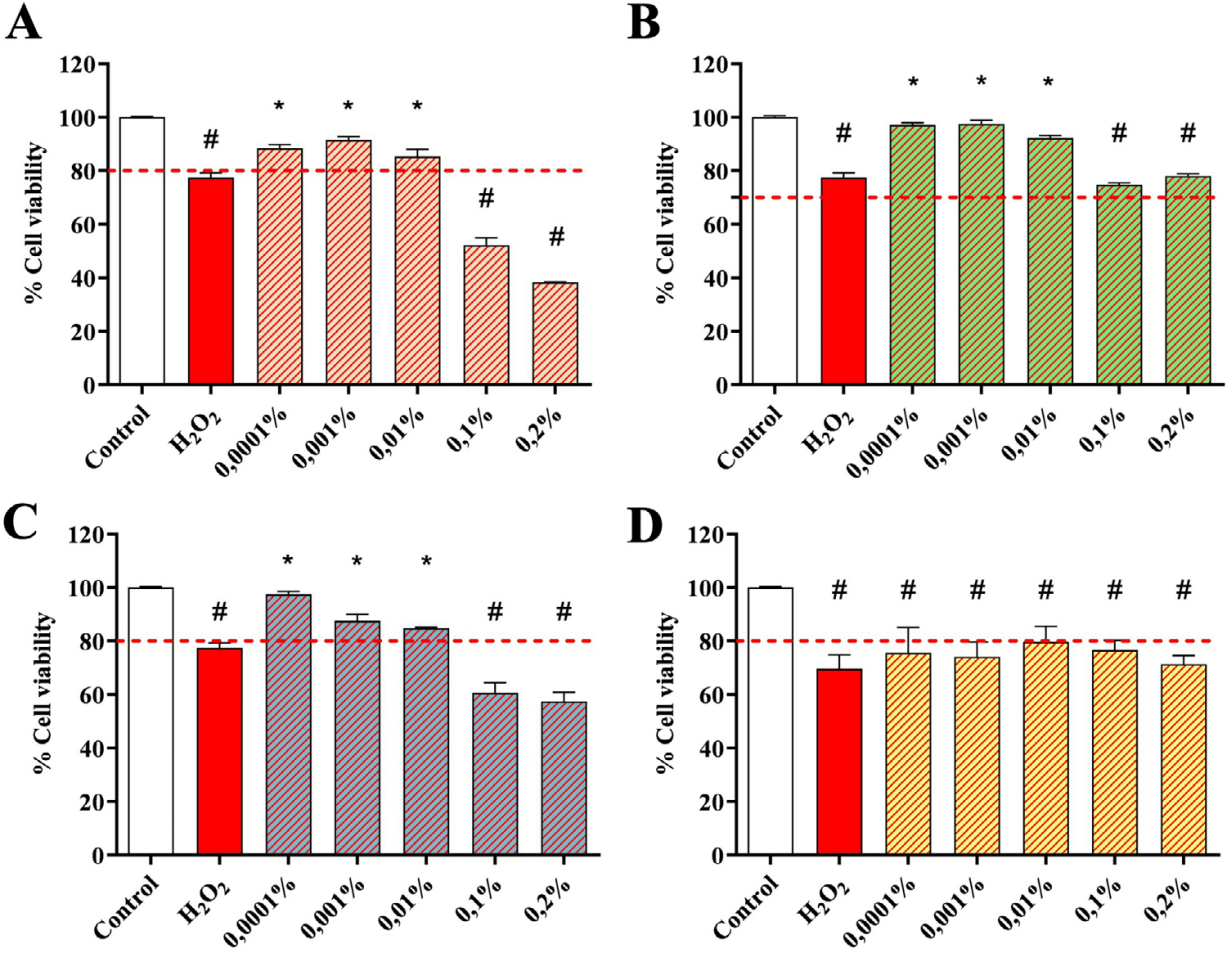
The anti‐oxidative potential of *L. angustifolia* essential oil (A), *L*. “Grosso” essential oil (B), *L*. “Super” essential oil (C), and commercial lavender essential oil (D) on SH‐SY5Y exposed to H_2_O_2_, as an oxidative stimulus. Data are presented as a percentage of cell viability compared to untreated cells used as a control. The red dotted line indicates the percentage of cell viability under which the treatments were considered cytotoxic. Data are shown as mean ± SEM of three replicates: *p < 0.05 vs H_2_O_2_; ^#^ *p* < 0.05 vs. control group.

In order to test the anti‐oxidative potential of the three hydrodistilled LEOs and to compare them with the commercial LEO, the SH‐SY5Y cell line was initially exposed to each of the four LEOs at concentrations ranging from 0.0001% to 0.2%, for 24 h. Subsequently, cells were treated with H₂O₂ 0.2 mM, used as an anoxidative stimulus. After a further 24 h, cell viability was determined by means of an MTT assay. Pre‐treatment with *L. angustifolia* (Figure 2A), L. “Grosso” (Figure 2B), and L. “Super” (Figure 2C) EOs at concentrations ranging from 0.0001% to 0.01% was able to significantly increase cell survival compared to the H_2_O_2_ treated group. Interestingly, the commercial LEO did not show a significant protective effect against the H_2_O_2_‐induced oxidative stress at any concentration (Figure 2D).

**FIGURE 3:**
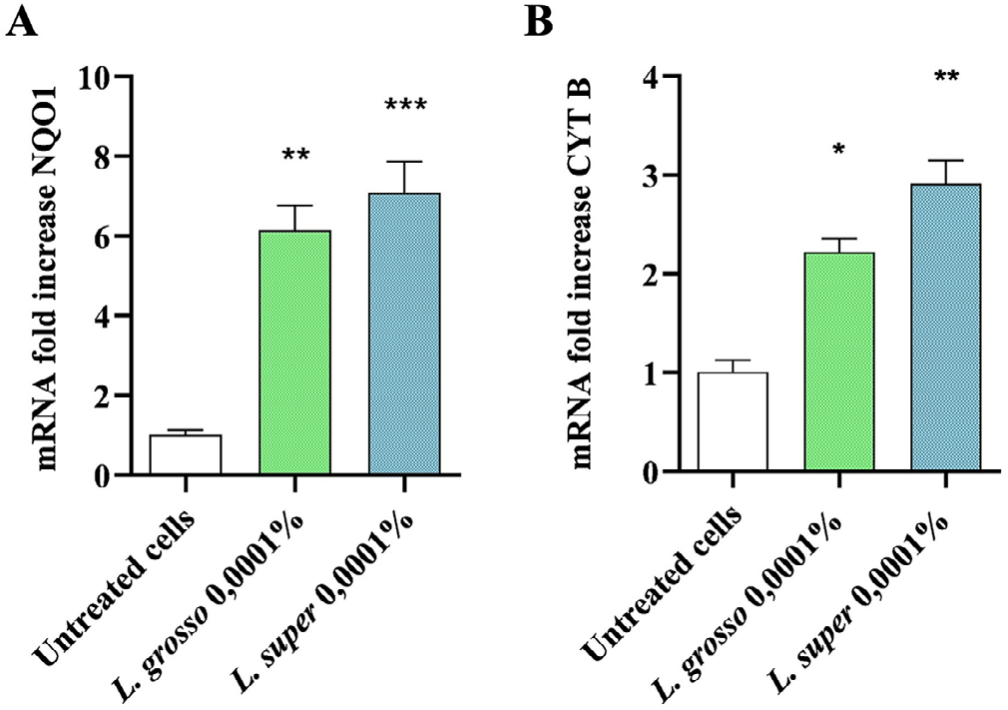
*L*. “Grosso” and *L*. “Super” essential oils increased the expression of two genes involved in antioxidant protection, NQO1 (A) and CYT B (B). SH‐SY5Y cells were treated with 0.0001% *L*. “Grosso” and *L*. “Super” essential oils for 12 h. The NQO1 and CYT B mRNA expression levels were then determined by real‐time polymerase chain reaction (PCR). glyceraldehyde‐3‐phosphate dehydrogenase (GAPDH) was used as a normalizer. Data are representative of three replicates and shown as mean ± SEM; *p < 0.05_;_ **p < 0.01; ***p < 0.001 versus control.

To deepen the understanding of the oxidative protection mechanism, SH‐SY5Y cells were treated with *L*. “Grosso” and *L*. “Super” EOs at 0.0001%, the lowest dose that resulted in protection against the H_2_O_2_ insult, for 12 h. The expression levels of two genes with antioxidant functions, namely NQO1 and CYT B, were assessed. This setup reflects the cellular conditions after the treatment with the LEOs and prior to H₂O₂ exposure, providing insight into how the EOs prime the cells for oxidative stress defense. As depicted in Figure 3, NQO1 and CYT B genes were not expressed in untreated cells, whereas treatment with 0.0001% *L*. “Super” and *L*. “Grosso” EOs resulted in a significant upregulation of both NQO1 (Figure 3A) and CYT B (Figure 3B). Additionally, *L*. “Super” demonstrated a more pronounced effect compared to *L*. “Grosso”.

## 3 Discussion

*L. angustifolia, L*. “Grosso”, and *L*. “Super” are plants belonging to the *Lavandula* L. genus and are mainly distributed in the Mediterranean area [16]. LEOs are characterized by unique aroma and therapeutic properties, including antimicrobial, anti‐inflammatory, and antioxidant abilities. Considering these aspects, lavender extracts and EOs have been used in traditional medicine since the Middle Ages [17]. Nowadays, given their wide range of uses, many LEOs are commercially available worldwide; however, their composition and properties, which can be influenced by their production, are not always assessed. In this study, three LEOs derived from *L. angustifolia, L*. “Grosso”, and *L*. “Super”, obtained by hydrodistillation, as well as a commercially available LEO, were compared for their phytochemical composition and biological properties. The chemical profiles of the three LEOs extracted via hydrodistillation resulted to be similar to the commercially available LEO in terms of main constituents; linalool and linalyl acetate represented the main identified compounds, in accordance also with the literature [18], while oxygenated monoterpenes were the prevalent class of compounds [11, 19–21]. Although the commercial LEO was characterized by a higher content of linalyl acetate, linalool, and beta‐caryophyllene compared to the ones obtained through hydrodistillation, it showed reduced levels of α‐terpineol and 4‐terpineol and the total absence of terpinolene and α‐pinene. These compounds are known to be biologically active; specifically, α‐terpineol has been shown to have anti‐inflammatory properties [22], while terpinolene has antioxidant activity [23]; α‐pinene and 4‐terpineol are characterized by anti‐inflammatory and antioxidant properties [24, 25, 26]. This synergistic effect among the various molecules present in EOs has been extensively studied in the literature [27]. Findings indicate that the overall effect is attributed to the entire phytocomplex and that even molecules present in small amounts play a significant role in biological activity [28]. This concept of the phytocomplex [29] helps explain why commercial EO, despite being rich in major components such as linalool and linalyl acetate, does not exhibit the same biological activity observed in hydrodistilled EOs.

Furthermore, it is worth mentioning that in the commercial LEO were detected traces of chemicals not found in any of the other three LEOs, like 2‐*p*‐menthen‐1‐ol, *cis*‐*p*‐menth‐2‐en‐1‐ol, and 1‐octen‐3‐ol. Among these molecules, only 1‐octen‐3‐ol has been reported in the literature and it has been associated with pro‐inflammatory effects and target mitochondria, with alterations in the bioenergetic and in the levels of antioxidant enzymes in *Drosophila melanogaster* [30, 31].

To evaluate their antioxidant effect, the four LEOs were tested in vitro on a human neuroblastoma cell line (SH‐SY5Y) at concentrations ranging from 0.0001% and 0.2%. The LEOs obtained through hydrodistillation did not show any cytotoxic effect, except at the highest concentrations. The commercial LEO did not reduce cell viability at any of the considered concentrations, as well. Regarding the antioxidant properties of the EOs, the hydrodistilled LEOs had a significant protective effect against the H_2_O_2_‐induced oxidative stimulus at concentrations ranging from 0.0001% to 0.01%.

However, the commercially available LEO did not show a protective effect at any of the used dosages. To have an in‐depth understanding of the antioxidant properties of the four LEOs, the expression levels of NQO1, involved in oxidative protection [32], and CYT B, a component of respiratory chain complex III [33], were evaluated in the SH‐SY5Y cell line after a 12‐h exposure to 0.0001% *L*. “Grosso” and *L*. “Super” EOs. These EOs were selected based on their low cytotoxicity and because only a few studies present in the literature focus on them. The results showed that both EOs significantly increased the expression levels of both genes compared to the non‐treated control, with *L*. “Super” outperforming *L*. “Grosso”. The results of the antioxidant activities obtained are congruent with other studies, which show *L. angustifolia, L*. “Grosso”, and *L*. “Super” extracts to have a significant scavenger‐like effect against free radicals, as tested with different chemical assays [5, 12, 34].

Several studies have also emphasized the antioxidant potential of *L. angustifolia* and lavandin EOs, demonstrating that their activity is influenced by variations in chemical composition as well as environmental factors, including soil composition, pH, and geographic location [35]. Specifically, Gavrić et al. [36] and Détár et al. [37] demonstrate that the antioxidant capacity of *L. angustifolia* and lavandin EOs can vary depending on the cultivar and the content of metabolites, such as flavonoids and, more broadly, phenolic compounds.

In conclusion, the EOs of *L. angustifolia, L*. “Grosso”, and *L*. “Super” all showed to have significant protection against oxidative stress caused by the H_2_O_2_ treatment; since the main compounds found in LEOs are known for their antioxidant properties [38, 39], the effect previously mentioned could be attribute to the cooperative synergistic effects of the chemical compounds in the EOs, as also reported in the literature [40]. The ratios and interactions of the compounds enable them to act as scavengers, intercepting free radicals. However, this ability is absent in the commercially purchased LEO, which showed no protective effects at any of the tested concentrations. This lack of activity could be attributed to the different composition of the EO, as evidenced by the GS‐MS results. We hypothesize that decreased levels of α‐terpineol and 4‐terpineol, the absence of terpinolene and α‐pinene, as well as the presence of additive compounds influence the antioxidant effects of the mixture, as also reported by Hosseini et al. [41]. These chemicals that are added during the manufacturing process can improve and exalt the fragrance of the EO, as well as its conservation for longer periods of time at the expense of their biological activities and natural genuineness, resulting in the sophistication of the product.

## 4 Materials and Methods

### 4.1 Plant Materials and EOs

*L. angustifolia, L*. “Grosso”, and *L*. “Super” EOs were kindly provided by the University of Pisa. The EOs were obtained from plants cultivated during the summer of 2018 in Tuscany (Bibbona, Livorno, Italy; 43°27′N. 10°58′E. 60 m a.s.l). The commercially available EO is made by the “VicTsing” company and was bought by an online retailer. A sample of each EO was preserved and cataloged at the Department of Molecular and Translational Medicine, Division of Pharmacology, University of Brescia.

### 4.2 Hydrodistillation

To obtain the EOs, 50 g of air‐dried aerial parts of each lavender were hydrodistilled by a Clevenger‐type apparatus for 2 h, as reported in the European Pharmacopoeia. The extracted oils were then conserved at 4°C and maintained far from any light sources until analyses.

### 4.3 GC‐MS Analysis

The chemical analysis was performed, in triplicates, by GC‐MS on EOs diluted to 5% with HPLC‐grade *n*‐hexane. The GC‐MS analysis was carried out with an Agilent 7890B gas chromatograph paired with an Agilent 5977B single quadrupole mass detector (Agilent Technologies, Inc., Santa Clara, CA) furnished with an Agilent HP‐5MS capillary column (30m x 0,25 mm; coating thickness 0,25 µm).

The analytical conditions were set as follows: the injector and transfer line temperatures at 220 and 240°C, respectively, oven temperature progression from 60 to 240°C at a 3°C/min rate, helium as carrier gas flowing at 1 mL/min with a split ratio of 1:25. The acquisition operated in full scan mode within the range of 30–300 m/z at 1,0 second scan time. The compound identification was based on the comparison of the sample's retention time and indices with authenticated samples and a series of n‐hydrocarbons. Additionally, computer‐assisted matching was used against both commercial [42] and lab‐developed mass spectra libraries, comprised of pure substances, known EO constituents, and MS literature data [43, 44, 45, 46].

### 4.4 Cell Culture and Treatments

The SH‐SY5Y cell line (human neuroblastoma cells) was cultured in cell culture medium composed of DMEM and Ham's F12 medium 1:1, supplemented with 10% fetal bovine serum, 0.5% L‐glutamine, 100 U/mL penicillin and 100 µg/mL streptomycin, at 37°C and 5% CO_2_. Cells were seeded at a density of 2 × 10^4^ cells/well in 96‐well plates. For the cytotoxicity test, cells were treated with the four LEOs at different concentrations (0.2%, 0.1%, 0.01%, 0.001%, and 0.0001%) for 48 h. Untreated cells were used as control.

The antioxidant activity was then tested by treating SH‐SY5Y cells with the four LEOs at the concentrations previously used for 24 h; cells were then exposed to the oxidant stimulus H_2_O_2_ 0.2 mM for a further 24 h. Untreated cells and cells treated solely with H_2_O_2_ 0.2 mM were used as controls.

### 4.5 Cell Viability

After the treatments, the viability was assessed by incubating SH‐SY5Y cells with 500 mg/mL of MTT for 90 min at 37°C; they were then lysed with DMSO and the absorbance was measured at 570 nm using an EnSight Multi‐mode Plate Reader (PerkinElmer, Waltham, MA, USA).

Data were expressed as a percentage of cell viability over the control group. The results were expressed as mean ± SEM.

### 4.6 Quantitative Real‐Time Polymerase Chain Reaction

To deepen the antioxidant potential of the two more promising LEOs, SH‐SY5Y cells were treated with *L*. “Grosso” and *L*. “Super” EOs at 0.0001% for 12 h. The expression of two genes involved in cellular protection against ROS, namely NQO1 (NAD(P)H quinone dehydrogenase 1) and CYT B (cytochrome B), was then evaluated. Specifically, cells were seeded at a density of 9 × 10^4^ cells/well in 24‐well plates, and total RNA was extracted following the TRIzol reagent protocol. Subsequently, 2 µg of total mRNA were reverse‐transcribed using a reaction mix consisting of 5 mM oligo (dT) primers, 10 U/mL M‐MLV reverse transcriptase, 1 mM dNTPs, 1 U/mL RNase inhibitor, 1X RT buffer. The reverse transcription reaction was performed in three steps: 10 min at 70°C, 2 min at 4°C, and 60 min at 37°C. ViiA7 Real‐Time Polymerase Chain Reaction (RT‐PCR) Detection System (Applied Biosystems, Foster City, CA, USA) was used for Real‐time PCR, for which were used 6 µL of SYBR Green Master Mix, 6 pmol of forward and reverse primers, and 2 µL of cDNA. The Real‐time PCR reaction steps were 10 min at 95°C, 40 cycles at 95°C for 15 s each, and finally at 60°C for 60 s. Primers sequences were CYT B (F: CCAGCTACCATGTCCCAGAT, R: TATGCCAGCTTCCGACTCTT) and glyceraldehyde‐3‐phosphate dehydrogenase (GAPDH) (F: GAGTCAACGGATTTGGTCGT, R: TTGATTTTGGAGGGA TCTCG). NQO1 QuantiTect primers were provided by Qiagen (Qiagen, Hilden, Germany). GAPDH was used as an endogenous reference. Quantitative RT‐PCR was performed with the ViiA7 RT‐PCR System (Applied Biosystems, Foster City, CA, USA) using the iQ SYBR Green Supermix method (Bio‐Rad Laboratories, Richmond, CA, USA) according to the manufacturer's instructions. The comparative Ct method was used for relative quantification. Data were presented as the fold change in target gene expression. GAPDH was used as a calibrator. The comparative Ct method was used for relative quantification. Data were presented as the fold change in target gene expression.

### 4.7 Statistical Analysis

The results are presented as mean ± standard error mean of three independent replicates. Data were analyzed using a one‐way analysis of variance test, followed by Dunnett's test for multiple comparisons. A *p*‐value < 0.05 was considered significant. Statistical analyses were performed using GraphPad Prism 9.0.0 (GraphPad Prism Software, San Diego, CA, USA).

## Author Contributions

Andrea Mastinu, Giulia Abate, Luisa Pistelli, and Guido Flamini were responsible for conceptualization. Ylenia Pieracci and Gianluca Angius contributed to the data collection. Eileen Mac Sweeney, Ylenia Pieracci, Vlad Sebastian Popescu, and Gianluca Angius conducted the analysis and produced the results and figures. Eileen Mac Sweeney, Vlad Sebastian Popescu, and Gianluca Angius drafted the manuscript. All the authors have read and approved the final manuscript.

## Conflicts of Interest

The authors declare no conflicts of interest.

## Data Availability

The data that support the findings of this study are available from the corresponding author upon reasonable request.
